# Unveiling the Phytochemical Diversity of *Pereskia aculeata* Mill. and *Pereskia grandifolia* Haw.: An Antioxidant Investigation with a Comprehensive Phytochemical Analysis by Liquid Chromatography with High-Resolution Mass Spectrometry

**DOI:** 10.3390/ph19010038

**Published:** 2025-12-23

**Authors:** Eduarda C. Amaral, Alan de A. Veiga, Juliana C. Atherino, Wesley M. de Souza, Diogo H. Kita, Francislaine A. Lívero, Gustavo da Silva Ratti, Simony R. B. Rosa, Ezilda Jacomassi, Lauro M. de Souza

**Affiliations:** 1 Instituto de Pesquisa Pelé Pequeno Príncipe, Av. Munhoz da Rocha 490, Curitiba 80035-000, Brazil; eduarda.amaral@aluno.fpp.edu.br (E.C.A.); alanwessels@hotmail.com (A.d.A.V.); diogokita@gmail.com (D.H.K.); 2 Faculdades Pequeno Príncipe, Curitiba 80230-020, Brazil; 3 Laboratory of Bacteriology, Mycology and Molecular Biology, Departamento de Análises Clínicas, Federal University of Paraná, Curitiba 80210-170, Brazil; ju.atherino@gmail.com (J.C.A.); wesleysouza@ufpr.br (W.M.d.S.); 4 Laboratory of Cardiometabolic Pharmacology, Departamento de Farmacologia, Federal University of Paraná, Curitiba 81530-000, Brazil; francislaine@ufpr.br; 5 Laboratory of Preclinical Research of Natural Products, Paranaense University, Umuarama 87501-390, Brazil; gustavoratti@gmail.com; 6 Departamento de Plantas Medicinais e Fitoterápicos na Saúde Primária, Universidade Paranaense—Unipar, Umuarama 87501-390, Brazil; simony.n@edu.unipar.br (S.R.B.R.); ezilda@prof.unipar.br (E.J.)

**Keywords:** ora-pro-nóbis, natural products, phenolic compounds, saponins, fingerprint analysis

## Abstract

**Background/Objectives:***Pereskia aculeata* and *Pereskia grandifolia* belong to the Cactaceae family, despite their foliar and woody stem characteristics. Both species are commonly known as Ora-pro-nóbis (derived from Latin, meaning “pray for us”), a name rooted in their historical use in colonial Brazil due to their nutritional value, particularly *P. aculeata*, which is frequently described as a high-protein food source. The goal of the present study was to compare these species based on phytochemical composition and antioxidant capacity. **Methods**: Both species were investigated for their chemical antioxidant properties (DPPH and phosphomolybdenum complex) and cellular anti-ROS activity using the CACO-2 cell line. A comprehensive phytochemical analysis was performed using LC-MS and GC-MS. **Results**: *P. aculeata* exhibited a more abundant content of phenolics and flavonoids, with greater structural variability in phenolic compounds and glycosylated flavonoids than *P. grandifolia.* Still, *P. aculeata* showed a more potent chemical antioxidant effect. By contrast, in *P. grandifolia*, a series of novel saponins was now discovered and characterized. In addition, the compounds from this species exhibited a greater cellular antioxidant activity than those of *P. aculeata*. Tryptophan-derived alkaloids, such as abrine (N-methyltryptophan), were present in both species, but hypaphorine only in *P. aculeata*. **Conclusions**: Both species of *Pereskia* exhibit potential health benefits, including distinct antioxidant activity, among other unexplored effects, given their significant variability in phytochemicals. These differences could be investigated in greater depth using combined LC-MS and GC-MS, thereby enabling more confident structural investigations of these natural compounds.

## 1. Introduction

The genus *Pereskia*, although belonging to the Cactaceae, exhibits remarkable morphological variation. Unlike other species in the Cactaceae, *Pereskia* spp. have woody stems and well-developed leaves that function as photosynthetic organs. They are predominantly distributed in tropical regions, especially in Central and South America, where they hold significant ethnobotanical relevance. Several species are traditionally used in folk medicine to treat various diseases; however, because of their high nutritional value, they are primarily consumed as food [1]. Investigations into the therapeutic and nutritional potential of unconventional food plants have intensified in recent decades, and some species from the genus *Pereskia* have garnered interest due to their rich chemical composition and promising pharmacological properties.

Among the species is the *Pereskia aculeata* Mill., which is the most investigated from this genus. Popularly known as “ora-pro-nóbis”, *P. acuelata* is valued for its nutritional content, with a high protein content (14–29% dry weight) and amino acids, which are the primary reason its leaves are consumed as a food source [1,2]. Other vitamins, minerals, and bioactive compounds are frequently associated with the benefits of consuming *P. aculeata*. Various phytochemical investigations have identified compounds in its leaves, including essential oils, phenolic compounds, derivatives of caffeic and coumaric acids; flavonoids and glycoside derivatives of quercetin, kaempferol, and isorhamnetin; carotenoids; mucilage composed mainly of arabinogalactan; and alkaloids [3,4,5,6,7].

In addition to its nutritional properties, the biological properties of *P. aculeata* were investigated, including potent antioxidant, anti-inflammatory, antinociceptive, wound-healing, antimicrobial, and selective cytotoxic effects against cancer cells, as well as beneficial effects on lipid metabolism and intestinal health [3,5,6,8,9,10,11,12]. Regarding antioxidant activity, extracts from the leaves of *P. aculeata* demonstrated a high capacity to eliminate free radicals and reduce oxidative damage in experimental models that evaluated their ability to reduce ferric iron and act as free-radical scavengers, thereby demonstrating their effectiveness in mitigating oxidative stress [13]. This property is of significant interest in preventing chronic diseases, such as diabetes, cardiovascular disease, and neurodegenerative disease, in which the imbalance between reactive oxygen species and endogenous antioxidant mechanisms plays a crucial role.

Although less investigated, *Pereskia grandifolia* Haw., also known as “ora-pro-nóbis-rosa”, is another important species from this genus. *P. grandifolia* is used in folk medicine to treat cancer, diabetes, hypertension, rheumatism, liver injuries, and as a diuretic; some potential properties were also investigated [1,14,15,16]. The phytochemical composition of *P. grandifolia* differed from that of *P. aculeata*, indicating the presence of terpenoids in the essential oil, as well as steroids such as β-sitosterol and stigmasterol, alkaloids [5,15,16,17], and phenolic compounds, including flavonoids such as quercetin and kaempferol, and glycosides [18].

Biological activities of *P. grandifolia* were also investigated, showing important properties including potent cytotoxicity/antiproliferative activity against cancer cell lines, remarkable hepatoprotective activity in a model of Metabolite-Associated Fatty Liver Disease, reversing steatosis, capacity of normalizing transaminases and lipid profile, and combating oxidative stress, in vitro anti-inflammatory activity and diuretic/hypotensive activity, possibly mediated by vasopressin reduction [15,17,19,20].

The Cactaceae family is a promising, albeit underexplored, source of bioactive compounds. Despite these species having been explored in pharmacological and phytochemical studies, few investigations provide structural details of plant phytochemicals that can support subsequent investigations. Moreover, the studied phytochemical profiles typically focus on the main compounds in extracts, which may not be directly related to their biological properties. Given the rich ethnobotany, potential biological activities, and distinct phytochemical compositions of *P. aculeata* and *P. grandifolia*, a detailed comparative analysis of these two species can help elucidate their therapeutic effects and potential pharmacological applications.

Therefore, the goal of this investigation was to provide, alongside antioxidant capacities, a comprehensive analysis of the phytochemical profiles of both species by performing detailed mass spectrometric fingerprinting. This technique generates a unique chemical profile, derived from the molecular mass converted to its respective ion (*m*/*z* = mass-to-charge ratio) for each compound, which can be fragmented in a controlled manner to produce individual spectra, also known as “fingerprints,” even from a complex sample like a plant extract. By combining this technique with chromatography, it is possible to measure all compounds simultaneously rather than isolating each component individually, enabling rapid provision of information on the plant’s chemical composition and highlighting their importance in phytochemical analysis.

## 2. Results

### 2.1. Chemical Antioxidant Activity

The plant extracts were partitioned into organic fractions (PA-But and PG-But) and aqueous fractions (PA-Aq and PG-Aq), which were submitted to antioxidant assays, and, considering the relevance of phenolic compounds, total flavonoids and polyphenols were quantified. The chloroform fractions from both species were not investigated because of their low solubility and high pigment content, which hindered assay performance.

The ability to reduce the DPPH reagent indicates the antioxidant potential of a plant extract. In this regard, the potential antioxidant is directly related to the extract composition. In our experiments, the PA-But fraction (butanolic fraction of *P. aculeata*) exhibited the most potent antioxidant activity, with an IC_50_ of 22 µg · mL^−1^, indicating a very strong antioxidant capacity. In contrast, the similar fraction from *P. grandifolia* had an IC_50_ of 53 µg·mL^−1^, which, although less effective than PA-But, is considered to have strong antioxidant capacity. Both fractions confirmed the antioxidant potential of these plant constituents, and the differences observed must be related to the phytochemical composition, since *P. aculeata* (PA-But) exhibited greater amounts of polyphenols (102 mg·g^−1^) and flavonoids (69 mg·g^−1^), compared to *P. grandifila* (PG-But), that had 28.8 and 40.8 mg·g^−1^, of total polyphenols and flavonoids, respectively.

The capacity to reduce the molybdenum ions also reflects the antioxidant potential, and, again, the fraction PA-But (*P. aculeata*) was better than PG-But (*P. grandifolia*), and both organic fractions were more potent than the aqueous fractions (Table 1). Notably, the aqueous fractions had considerably lesser antioxidant effects than the organic fractions, and this could be well explained in terms of total polyphenols and flavonoids retained in the aqueous fractions, which were lower than those in the organic counterparts. This also explains why the PA-But fraction exhibits the highest chemical antioxidant activity, as it contains the highest amount of phenolic compounds (Table 1). At first glance, this suggests that *P. aculeata* has higher levels of chemical antioxidant compounds than *P. grandifolia*.

### 2.2. Effects of PA-But and PG-But on Intracellular ROS Production

For the cell antioxidant evaluation, CACO-2 cells were stimulated to generate reactive oxygen species (ROS) by incubation with the chemotherapeutic agent 5-fluorouracil (5-FU) and monitored using the ROS-sensitive probe 2,7-dichlorofluorescein diacetate (DCFH-DA). The samples were chosen based on the antioxidant activity observed in the chemical assays; therefore, the PA-But and PG-But were used in this assay.

**Figure 1:**
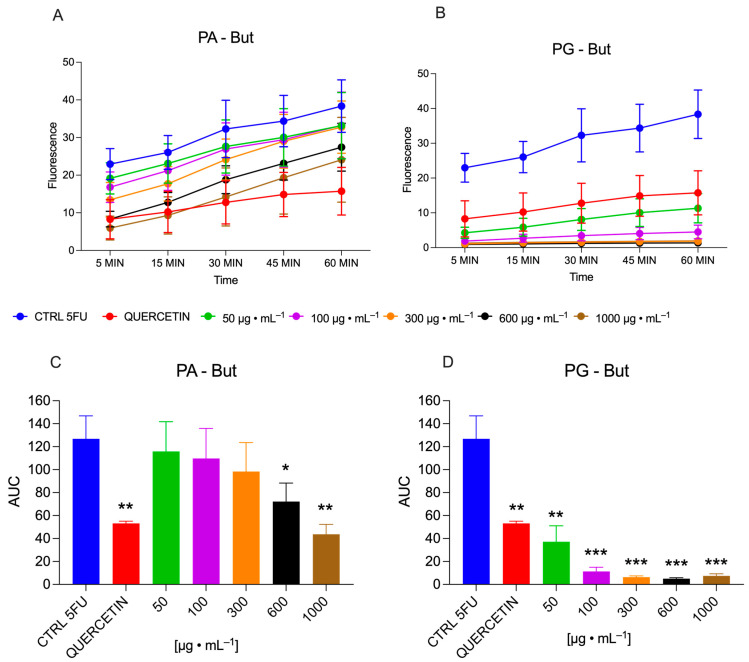
Cellular antioxidant activity was assessed as the ability of the *P. aculeata* and *P. grandifolia* fractions to inhibit the intracellular ROS, stimulated with 5-fluorouracil. The fractions PA-But (**A**) and PG-But (**B**) were monitored for 60 min, with PG-But showing greater anti-ROS activity. This was confirmed by the AUC calculated from individual concentrations, demonstrating that the PA-But fraction (**C**) was effective only at higher concentrations (600 and 1000 μg·mL^−1^). In contrast, the PG-But fraction exhibited a strong antioxidant effect at the lowest tested concentration, and achieved decreases greater than 90% at the higher concentrations (**D**). The asterisks indicate the confidence level, as follows: * *p* < 0.05, ** *p* < 0.01, *** *p* < 0.001.

On the one hand, the PA-But fraction had the highest chemical antioxidant activity; on the other hand, this was not observed in the cellular assay, where the PG-But fraction exhibited the highest capacity to prevent the ROS production, whereas the PA-But fraction had no activity in most concentrations tested, but the higher ones with a modest reduction in cell ROS, over the incubation time (60 min) (Figure 1A). By contrast, the PG-But fraction exhibited a strong capacity to reduce cellular ROS even at the lower concentration tested (50 μg·mL^−1^), which was superior to that observed with the quercetin control at 5 μg·mL^−1^ and achieved near-zero levels at the higher concentrations, along the incubation period (Figure 1B).

The overall ROS production was quantified as the area under the curve (AUC), converted to total cellular antioxidant activity, and normalized to percent ROS inhibition. No statistically significant differences were observed between the control (5-FU) and the PA-But fractions at concentrations of 50, 100, and 300 μg·mL^−1^; however, a modest decrease in cell ROS content was observed at 600 and 1000 μg·mL^−1^, corresponding to 32% and 45%, respectively (Figure 1C). A completely different behavior was observed with the PG-But fraction, which, at lower concentration (50 μg·mL^−1^), exhibited superior antioxidant capacity than the quercetin control (5 μg·mL^−1^), inhibiting cell-ROS by 66.7%, compared with 58.3% for quercetin. PG-But fraction at a concentration of 100 μg·mL^−1^ reduced overall ROS production by 86% compared with the control (5-FU), whereas at higher concentrations, cell ROS were decreased by more than 90%, with no statistically significant differences among them (Figure 1D).

### 2.3. Phytochemical Composition of Fractions PA-But and PG-But

**Figure 2:**
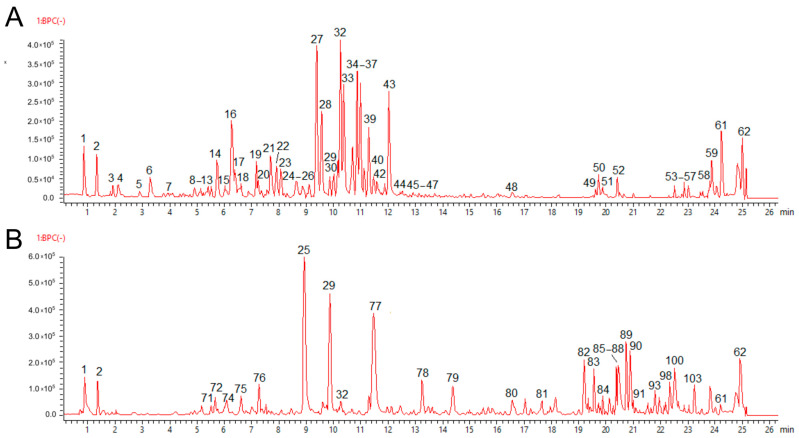
Phytochemical analysis of the fractions from *Pereskia aculeata* (**A**) and *Pereskia grandifolia* (**B**). Distinct chemical constituents are observed in the chromatograms of the PA-But and PG-But fractions. The peak numbers correspond to the compounds further described in the text and Table 2.

The fractions PA-But and PG-But were investigated for their phytochemical composition. Both were analyzed by LC-MS/MS, in positive and negative ionization modes. The extracts from both plants exhibited distinct chromatographic profiles (Figure 2A,B), confirming that their phytochemical compositions differed markedly. The compounds were observed as protonated [M+H]^+^ or deprotonated [M-H]^−^ ions, as well as conjugated with adducts, such as K^+^, Na^+,^ NH_4_^+^, Cl^−^, or HCOO^−^. The fragmentation spectra were compared with literature and spectral databases, allowing the identification of most of these fraction components, including potential novel compounds for these species.

*Pereskia aculeata*—this species has been better investigated than the *P. grandifolia* and, therefore, several compounds were previously described [3,4,5,21]. Despite this, our analysis identified some unreported compounds in the organic fraction of the leaf extract. Besides common compounds, such as disaccharides (e.g., sucrose) appearing at *m*/*z* 381.075 [M+K]^+^, the positive ionization mass spectrometry (MS) revealed a series of nitrogen-containing compounds, typical of alkaloids. Although some of these compounds were not identified, others were tentatively characterized based on their CID-MS spectra.

Common plant compounds, as (**1**) disaccharide (e.g., sucrose), (**2**) citric acid, (**6**) protocatechuic acid, (**7**, **12**) caffeoyl glucoside, (**13**) *p*-coumaroyl-glucoside, (**16**) caffeic acid, and (**24**) *p*-coumaric acid, were detected in this fraction (Table 2).

**Figure 3:**
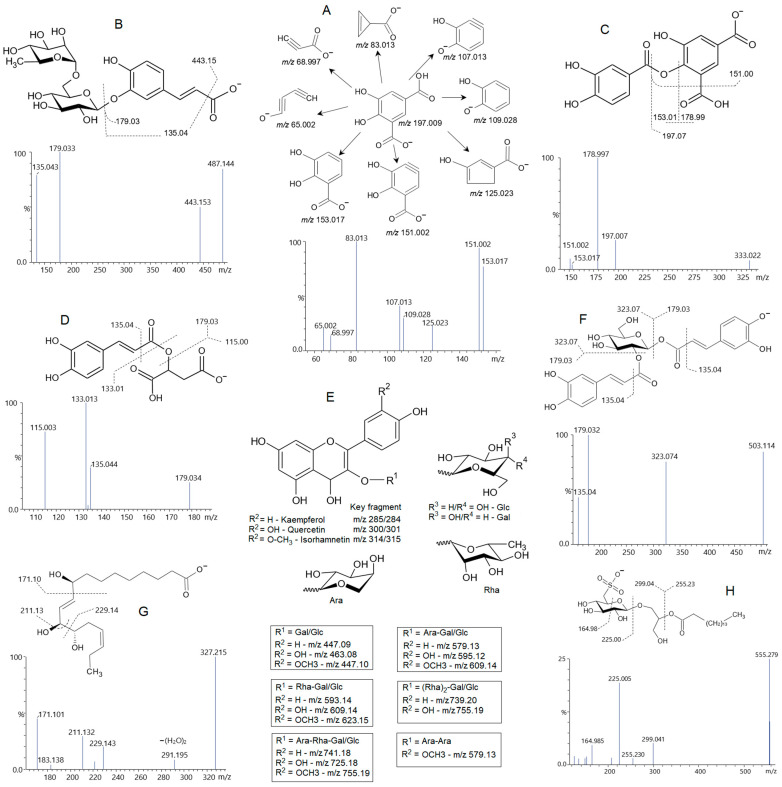
Proposed structure and mass spectra (MS^2^) data obtained in negative ionization. (**A**) Compound **11**, formed by a caffeic acid attached to a rutinose. (**B**,**C**) Structures for compounds **4** and **17**, based on mass spectra, are consistent with these depicted compounds. (**D**) Chemical structure of the compound **21**, tentatively identified as caffeoyl malic acid. (**E**) Structure and diversity of flavonol glycosides, composed of kaempferol, quercetin, and isorhamnetin, attached by mono- or oligosaccharides formed by Ara (arabinose), Rha (rhamnose), Gal (galactose), and Glc (Glucose). (**F**) Structure of compound **47**, consistent with a 1,3-dicaffeoylglucose. (**G**) A representative structure of trihydroxy-octadecadienoic acid, as found for compounds **49**–**51**, and the MS data, consistent with the structure. (**H**) The structure of a sulfoquinovosylmonoacylglycerol, compound **62**, the mass spectrum contains key fragments (i.e., *m*/*z* 225.005) that support this structure.

Compound **4**, at *m*/*z* 197.008 [M-H]^−^, with a fragment at *m*/*z* 153.018, suggests it is a protocatechuic acid derivative. The neutral loss (NL) of 43.99 Da indicates the presence of another carboxylic acid in this structure, which was lost as CO_2_, suggesting a structure like a dihydroxybenzene-dicarboxylic acid (Figure 3A). Compounds **5** and **15**, at *m*/*z* 315.107 [M-H]^−^, were identified as hydroxytyrosol-glucoside isomers, as observed through the fragment at *m*/*z* 153.054, consistent with the hydroxytyrosol moiety (NL of 162.053 Da), typical of a hexose residue [22]. The compound **8**, at *m*/*z* 175.060 [M-H]^−^, was consistent with isopropylmalic acid, with similar fragments (Table 2), as described in PubChem.

Compound **11**, *m*/*z* 487.144 [M-H]^−^, had similar fragments of caffeoyl-glucosides (i.e., *m*/*z* 179.034 and 135.045 from caffeoyl moiety). The simple caffeoyl-glucosides (as in **13**) appeared at *m*/*z* 341.085, but the compound **11** had 146.058 Da higher than this typical compound, and this difference is consistent with a rhamnosyl residue, suggesting a disaccharide, like rutinose, linked to caffeic acid. The compound swertiamacroside has previously been reported from other plants, such as *Fagopyrum esculentum* [23], which contains a rutinose moiety attached to caffeic acid via an ester linkage. Despite this similarity, the compound **11** yielded a fragment at *m*/*z* 443.153, corresponding to the loss of CO_2_ (NL 43.98 Da). Therefore, unlike Swertiamacroside, the compound **11** has a rutinose (or isomer) linked to the hydroxyl group of caffeic acid, instead via ester linkage (Figure 2B).

The compound **17** appeared at *m*/*z* 333.022 [M-H]^−^ and displayed fragments consistent with compound **4** (dihydroxybenzene-dicarboxylic acid), specifically that one at *m*/*z* 197.008, indicating the same core compound. The neutral loss (NL) of 136.014 Da is consistent with a protocatechuic residue attached to dihydroxybenzene-dicarboxylic acid. Fragments at *m*/*z* 178.997 (dehydrated residue of *m*/*z* 197.008), 153.017, and 151.002 support this statement (Figure 3C). Although the MS fragments are robust with the proposed structure, details of where these groups are attached could not be inferred.

Compound **20**, *m*/*z* 567.261 [M+HCOO]^−^, and fragments at *m*/*z* 521.257 (loss of adduct), 389.217 (loss of pentosyl residue), 293.085, and 161.045 (from hexosyl residue). This structure is formed by a disaccharide linked to a terpenoid, as the structure of Alangionoside B or its isomer, previously described in *Alangium premnifolium* [24].

Compound **21**, at *m*/*z* 295.044 [M-H]^−^, gave the fragments from caffeic acid (i.e., *m*/*z* 179.033, 135.044); and those consistent with malic acid (i.e., *m*/*z* 133.013 and 115.003), indicating a caffeoyl malic acid (Figure 3D), as previously reported in other plants [25]. Compound **22**, *m*/*z* 359.096 [M+HCOO]^−^, with a fragment at *m*/*z* 151.038, was consistent with a methoxybenzoyl-hexoside, and compound **23**, *m*/*z* 371.096 [M-H]^−^, seemed to be formed from a benzoic acid derivative, since the fragment at *m*/*z* 121.028 was consistent with benzoic acid.

Flavonoids and their glycosides were well reported in *P. aculeata* [21]. In the fraction PA-But, we also found a series of these compounds, mainly formed by quercetin, kaempferol, and methylquercetin, which had been previously identified as isorhamnetin [3,21], as aglycones. Monosaccharides identified by GC-MS in this fraction were: rhamnose (14%), arabinose (1.5%), galactose (35.2%), and glucose (49.3%). Characteristically, the fragments from flavonol glycosides yield the aglycone ions via a regular heterolytic cleavage, but a homolytic cleavage, yielding a radical ion, is frequently found, mainly in flavonol 3-*O*-glycosides [26,27]. Herein, the main fragment-ions from aglycones appeared as radical ions, at *m*/*z* 300.025 from quercetin, *m*/*z* 284.031 from kaempferol, and *m*/*z* 314.041 from isorhamnetin, indicating the possible glycosylation site (Figure 3E).

Isomers of these flavonol glycosides are relatively common, usually resulting from a Glc or Gal unit linked to the aglycone [28]. Similarly, we identified isomers for almost all the flavonol glycosides reported herein. Compounds **25** and **26** yielded negative ions at *m*/z 755.199 [M-H]^−^, with a main fragment at *m*/*z* 300.025, which were identified as quercetin-di-rhamnosyl-hexosides. Those **27** and **28** appeared at *m*/*z* 741.184 [M-H]^−^, the aglycone fragment gave *m*/*z* 300.025, indicating the presence of quercetin, and the fragment at *m*/*z* 609.140, from both, confirmed the loss of a pentose residue. Considering the presence of a single pentose (arabinose) from GC-MS analysis, the compounds **27** and **28** were assumed to be quercetin-arabinosyl-rhamnosyl-hexosides (Table 2), as previously found in plants [21].

Compound **29**, at *m*/*z* 739.204 [M-H]^−^, gave an aglycone-fragment at *m*/*z* 284.031, which is relatively common for kaempferol-di-rhamnosyl-hexosides [26,28], despite not being found previously for *P. acuelata*. Compounds **30** and **31**, at *m*/*z* 595.127 [M-H]^−^, were consistent with quercetin-arabinosyl-hexosides; and **32**, *m*/*z* 609.142 [M-H]^−^, consistent with rutin, as previously described [3]. Isomers **33** and **34**, *m*/*z* 725.188 [M-H]^−^, were identified as kaempferol-arabinosyl-rhamnosyl-hexosides. Although they share similar glycan moieties, as in **27** and **28**, these compounds have not been reported in *P. aculeata*. The last quercetin hexosides (**35** and **37**) were found at *m*/*z* 463.084 [M-H]^−^, and free quercetin (**48**) at *m*/*z* 301.033 [M-H]^−^.

Compound **36,** at *m*/*z* 755.199 [M-H]^−^, although an isomer of **25**/**26**, gave the aglycone fragment at *m*/*z* 315.048/314.041; therefore, consistent with isorhamnetin-arabinosyl-rhamnosyl-hexoside, as previously reported [3]. Kaempferol diglycosides were found at *m*/*z* 579.131 [M-H]^−^ (**38**) and *m*/*z* 593.147 [M-H]^−^ (**39**), being composed of arabinosyl-hexoside and rhamnosyl-hexoside, respectively; and kaempferol-hexoside (**42**) was found at *m*/*z* 447.090 [M-H]^−^. Similarly, isorhamentin diglycosides were detected at *m*/*z* 609.141 [M-H]^−^ (**41**) and *m*/*z* 623.157 [M-H]^−^ (**43**), featuring the same glycan moieties as compounds **38** and **39**. Two isorhamnetin monoglycosides (**44** and **45**) were also found at *m*/*z* 477.100 [M-H]^−^. Another glycoside (**46**) was found at *m*/*z* 579.132 [M-H]^−^, with main fragments at *m*/*z* 315.048/314.041, indicating the presence of two arabinose units attached to isorhamnetin. This compound was previously reported in *P. aculeata* [21].

The compound **47**, *m*/*z* 503.116 [M-H]^−^, gave fragments at *m*/*z* 179.033 and 135.044 consistent with caffeic acid, and a fragment at *m*/*z* 323.074 produced by the loss of a caffeic acid residue. The MS data for this compound are consistent with a dicaffeoyl hexose (Figure 3F). Although this is the first report of this compound in *P. aculeata*, a similar compound has been reported in *Balanophora japonica* [29].

Three intriguing isomers (**49**, **50**, **51**) were found at *m*/*z* 327.215 [M-H]^−^. These compounds were initially considered contaminants, but a thorough investigation identified them as trihydroxy-octadecadienoic acid (Figure 3G). Although the positions of hydroxyl groups and double bonds could not be resolved, this finding is relevant to this plant, as this class of fatty acids exhibits pharmacological properties, including antiatherosclerotic effects and antiatherogenic activity [30].

To our knowledge, saponins have not been reported in *P. aculeata*. Therefore, two novel saponins (**52**, **53**) were found in this fraction, at *m*/*z* 955.483 and 793.431 [M-H]^−^, respectively. These compounds were identified as NH_4_ adducts in positive-ion mode, with low-abundance intact ions. However, the main ion appeared at *m*/*z* 439.351, consistent with an in-source fragmentation that produced a dehydrated residue of oleanolic or ursolic acid. Therefore, compound **52** was identified as a saponin triglycoside, whereas **53** was a diglycoside composed of uronic acid (glucuronic or galacturonic acid) and hexoses (galactose or glucose) attached to the triterpenic acid.

Regardless of the chloroform partition, polar lipids were also detected in PA-But, primarily as lyso-lipids (monoacyl). They appeared as digalactosyl-monoacylglycerol (**54**, **56**) at *m*/*z* 721.350 [M+HCOO]^−^, the loss of adduct yielded *m*/*z* 675.355 and diagnostic fragments of *m*/*z* 277.216, characteristic of linolenic acid (C18:3), *m*/*z* 415.142 and 397.132 resulting from the digalactosyl-glycerol [31]. The isomers can be explained by the acyl linkage site (i.e., *sn*-1 or *sn*-2). Another digalacotsyl-monoacylglycerol (**59**) was found at *m*/*z* 699.375 [M+HCOO]- with similar polar head fragments as in **54**/**56**, but that one at *m*/*z* 255.231, consistent with palmitic acid (C16:0).

Lipids **55** and **57** were identified as a lyso-phosphatidylinositol C18:3 (*l*-PI), appearing at *m*/*z* 593.268 [M-H]^−^, with diagnostic fragments at *m*/*z* 277.216, from C18:3; *m*/*z* 241.010, from inositol phosphate residue, and *m*/*z* 152.994 from glycerol phosphate residue. The isomers, again, derive from the ester linkage site. Another *l*-PI (**58**) was found at *m*/*z* 595.284 [M-H]^−^, with similar fragments (Table 2), except for the presence of the one at *m*/*z* 279.231, indicating the presence of a linoleic acid (C18:2) in this structure. The lipid **61**, at *m*/*z* 571.284 [M-H]^−^, also gave similar fragments, but that at *m*/*z* 255.231 was consistent with palmitic acid (C16:0), indicating a *l*-PI C16:0.

The last lipid (**62**) in negative ionization was found in PA-But at *m*/*z* 555.280 [M-H]^−^, being identified as a sufoquinovosylmonoacylglycerol-C16:0 (SQMG) based on diagnostic fragments at *m*/*z* 299.041, from the sulfoquinovosylglycerol residue, and *m*/*z* 225.005, from the sulfoquinovosyl residue [31]. The fragment at *m*/*z* 255.230 confirmed the presence of palmitic acid (Figure 3H).

The fatty acids found in these lipids, herein described, were confirmed by GC-MS analysis. The fatty acids were analyzed as FAME derivatives and quantified by peak area, as relative abundance. Therefore, for PA-But, the fatty acid composition was: palmitic acid (C16:0) 45%, stearic acid (C18:0) 7.5%, linoleic acid (C18:2) 11.2%, and linolenic acid (C18:3) 36.3%.

**Figure 4:**
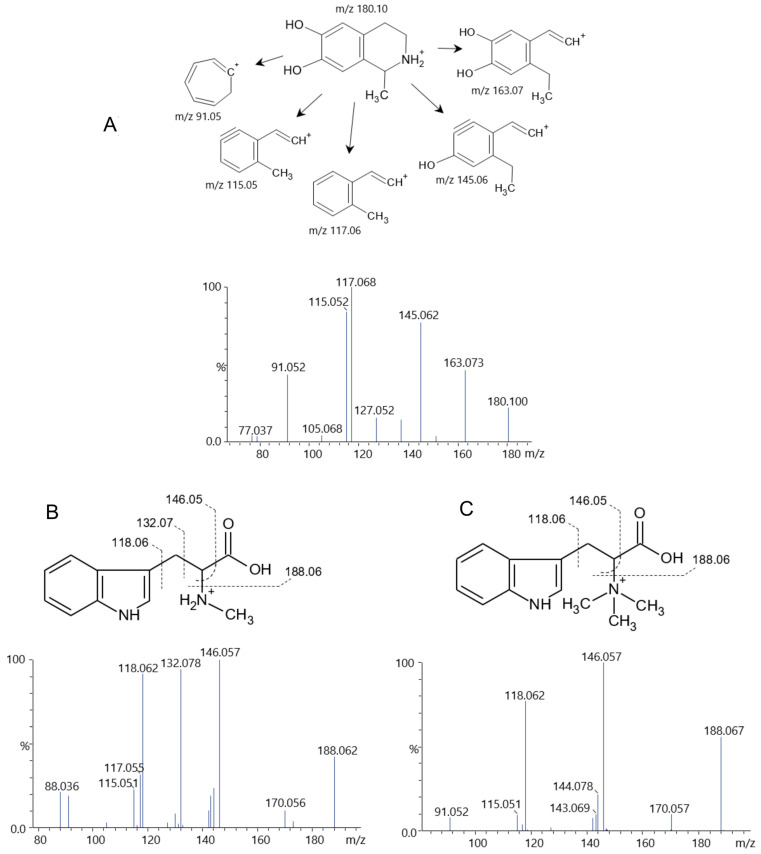
(**A**) Isomeric compounds **63**/**64** with similar mass spectra consistent with the structure of the isoquinolic alkaloid salsolinol. Structures and mass spectra of abrine (**B**) and hypaphorine (**C**), consistent with compounds **67** and **69**, were tentatively identified in *P. aculeata*.

Some compounds were best detected, albeit not exclusively, in the positive-ionization mode due to the presence of basic nitrogen. Two compounds (**63**, **64**) yielded *m*/*z* 180.100 [M+H]^+^, with predicted molecular formula C_10_H_13_NO_2_, with fragments at *m*/*z* 163.073 (-NH_3_), 145.062 (-NH_3_, -H_2_O), 117.068, 115.052, and 91.052 (tropylium ion). Some compounds, such as N-acetyltyramine, phenacetin, and salsolinol, share the same molecular formula; the literature indicates that salsolinol exhibits the same fragmentation profile as observed here (Figure 4A). Nevertheless, these compounds appeared in PA-But as isomers, and another compound that matches these characteristics is homophenylalanine, which has not been reported from plants but has been found in the cyanobacterium *Nostoc punctiforme* [32]. Additional analyses are necessary to confirm this structure.

Phenylalanine (**65**) at *m*/*z* 166.084 [M+H]^+^ and tryptophan (**66**) at *m*/*z* 205.095 [M+H]^+^ were also found and confirmed by their fragments (Table 2). Two other compounds exhibited a fragmentation profile similar to that of tryptophan. The compound **67**, at *m*/*z* 219.110 [M+H]^+^, 15 Da higher than tryptophan is consistent with abrine (N-methyl-tryptophan), previously found in *P. aculeata* [6], since the fragment at *m*/*z* 188.068, also found in tryptophan, indicate a loss of mathylamine (CH_3_NH_2_), confirmed the N-methyl group, and the fragment at *m*/*z* 118.063, from indole ring, are consistent with abrine (Figure 4B) [33]. The compound **68**, at *m*/*z* 247.141, was 42 Da higher than tryptophan, which could indicate the presence of a propyl radical bonded to the alpha-nitrogen or a betaine (trimethylamine) group. The fragmentation profile was similar to tryptophan and abrine, appearing at *m*/*z* 188.068, 146.057, 118.063, consistent with hypaphorine (Figure 4C) [34], which was not previously reported in the *Pereskia* genus.

Aminated lipids were also found in positive ionization (Table 2), as compound **69**, at *m*/*z* 318.296 [M+H]^+^, and fragments at *m*/*z* 300.285, 282.274, 264.264, yielded after a sequential molecular dehydration, which was consistent with phytosphingosine [35]. The compound **70**, *m*/*z* 476.333, was consistent with lyso-phosphatidylcholine (C16:0), with the diagnostic fragment appearing at *m*/*z* 184.069 [36].

*Pereskia grandifolia*—This species has been less investigated than *P. aculeata*, but it has attracted attention due to its potential, which has been explored more recently. The phytochemicals were poorly studied. However, some phenolics and alkaloids were previously reported [5,15,16,17,18].

In our LC-MS analysis of the fraction PG-But, we tentatively identified different compound classes, some of which were similar to those found in *P. aculeata*. Still, the phytochemical composition of the two species is quite distinct (Figure 2B). Some similar compounds were identified, including salsolinol (or its isomer) (**63**, **64**), abrine (**67**), and *p*-coumaroyl glucose (**16**). Flavonol glycosides were less abundant, with only four structures being found: quercetin-di-rhamonosyl-hexoside (**25**, **26**), rutin isomer 1 (**32**), kaempferol-di-rhamonosyl-hexoside (**29**), and rutin isomer 2 (**32**). Similar lipids were also found, such as digalactosyl-monoacylglycerol (**56**), lyso-phosphatidylinositol (**61**), and SQMG (**62**) (Table 2). Fatty acids were confirmed by GC-MS analysis, giving similar results as in PA-But: C16:0 47.5%, C18:0 10.8%, C18:2 4.2%, and C18:3 37.5%.

Terpenes glycosides were found in PG-But; these compounds were tentatively identified based on MS spectra and literature data. Compounds **71** and **73** appeared at *m*/*z* 527.229 [M+HCOO]^−^, whereas compound **72** appeared at *m*/*z* 395.188 [M+HCOO]^−^. Compound **72** yielded fragments at *m*/*z* 349.185 due to the loss of adduct, and *m*/*z* 179.055, consistent with a hexose, suggesting a structure similar to distyloside A [37]. Moreover, compounds **71** and **73** were 132.041 Da higher than **72**, strongly suggesting the presence of a pentose, as supported by fragments at *m*/*z* 149.042. Fragments at *m*/*z* 349.186 and 179.055 confirmed their similarity to compound **72**. Therefore, compounds **71** and **73** were classified as isomers, sharing the distyloside A core (**72**) but differing in the attachment of an additional pentose. A similar disaccharide was previously described for distyloside B; however, in this case, the aglycone differs [37]. The aglycone moieties should be further investigated to confirm their identity.

Compound **75**, *m*/*z* 447.146 [M+HCOO]^−^, yielding fragments at *m*/*z* 401.142 (loss of adduct), *m*/*z* 269.100, 193.048, 161.043. The latter could be from a dehydrated hexose residue, indicating a glycoside. Although these fragments were poorly understood, a benzyl β-primeveroside [38], or isomer, could be considered. The compound **76**, at *m*/*z* 431.188 [M+HCOO]^−^, with deprotonated ion at *m*/*z* 385.183 (loss of adduct), was interpreted as a terpenoid glycoside, since it produces fragments at *m*/*z* 223.133, 205.122, consistent with the aglycone and *m*/*z* 161.044 from a hexose residue. Several possible isomers were identified during the search for the molecular formula (C_19_H_30_O_8_) in the lotus. naturalproducts database, such as roseoside. Therefore, further purification and analysis are necessary to obtain more detailed information.

An unknown alkaloid (**77**) was found at *m*/*z* 262.070 [M-H]^−^, with a fragment at *m*/*z* 218.080, corresponding to an NL of 43.99 Da, consistent with the loss of CO_2_, typical of carboxylic acids. The fragment at *m*/*z* 200.070 (NL 18.01 Da) indicates the loss of a water molecule from the fragment *m*/*z* 218.080; and the fragment at *m*/*z* 146.059 could originate from a hydroxydihydroquinoline group, as in isoquinoline alkaloids.

Compounds **78** and **82** (*m*/*z* 691.255 and 675.261, respectively) appeared to share the same carbon backbone, but **78** contains a hydroxyl group that **82** lacks. Both loose acetyl groups with similar fragments, but the correct structure could not be assigned. Compound **79**, *m*/*z* 521.125 [M-H]^−^, was partially identified as a benzoic and protocatechuic acid derivative, since it produces fragments at *m*/*z* 152.010, indicating a negative radical ion from protocatechuic acid and *m*/*z* 121.027, consistent with benzoic acid.

**Figure 5:**
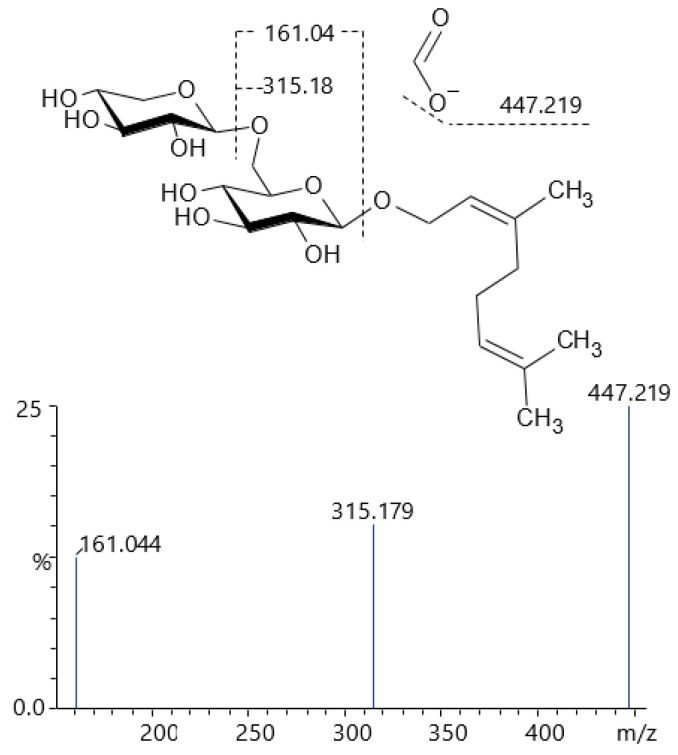
Proposed structure for compound **80** and its mass spectrum, showing strong correlation with geranyl β-primeveroside.

The compound **80**, *m*/*z* 493.224 [M+HCOO]^−^, was identified as a diglycoside, since it produced a fragment at *m*/*z* 447.219 (loss of adduct), followed by *m*/*z* 315.179, loss of 132.042 Da, consistent with a pentosyl residue, and a fragment from the hexose residue was found at *m*/*z* 161.044. This compound is consistent with a geranyl-pentosyl-hexoside (Figure 5), such as geranyl β-primeveroside [39].

Different from *P. aculeata*, in this fraction from *P. grandifolia*, a greater variability of triterpenoid saponins was found. In most of them, the aglycone is consistent with ursolic or oleanolic acids. LC-MS analysis of aglycones confirmed the presence of ursolic or oleanolic acid, but the chromatographic system could not resolve them to determine whether both were present. These positive ions were produced as ammonium adducts [M+NH_4_]^+^, which exhibited a strong tendency to undergo in-source fragmentation, yielding the ion at *m*/*z* 439.351 for most saponins, consistent with a dehydrated ursolic/oleanolic acid [40]. Additional potential aglycones were detected and will be discussed further.

Saponins have been poorly reported for *Pereskia* spp., but in 1974 [41], an oleanolic saponin was identified that contained a trisaccharide composed of glucuronic acid and glucose, structurally similar to those found herein. The monosaccharide composition, as determined by GC-MS, confirmed a high concentration of glucose (81.5%). However, galactose appeared at a concentration of 16.7%. Although this amount of galactose could arise from other glycosides, such as flavonols, we cannot exclude the possibility that it is present in the saponins, which could explain the presence of several isomers. Other monosaccharides appeared at low concentrations; rhamnose was only 1.8%, and arabinose was seen in less than 1%.

Compound **85**, at *m*/*z* 1117.533 [M-H]^−^, was consistent with soponin tetraglycoside, composed of three hexose units, as observed by sequential losses of 162/180 Da yielding fragments at *m*/*z* 955.490, 775.416, 613.368. A fragment at *m*/*z* 179.055 is consistent with hexose. A fragment at *m*/*z* 569.380 was not consistent with known aglycones, but the fragment at *m*/*z* 613.368 was consistent with ursolic/oleanolic acid linked to a dehydrated residue of uronic acid (e.g., glucuronic or galacturonic acid). The fragment at *m*/*z* 775.416 was 180 Da less than the ion at *m*/*z* 955.490, because it underwent a dehydration-like cleavage, supporting the statement that *m*/*z* 613.368 derives from the aglycone linked to a uronic acid residue. Therefore, the compound **85** was partially characterized as a ursolic/oleanolic acid trihexosyl glucuronide (Table 2). Most other saponins exhibited similar structures, differing only in glycosidic chains that included tri-, di-, and monosaccharides, as well as in isomeric forms.

**Figure 6:**
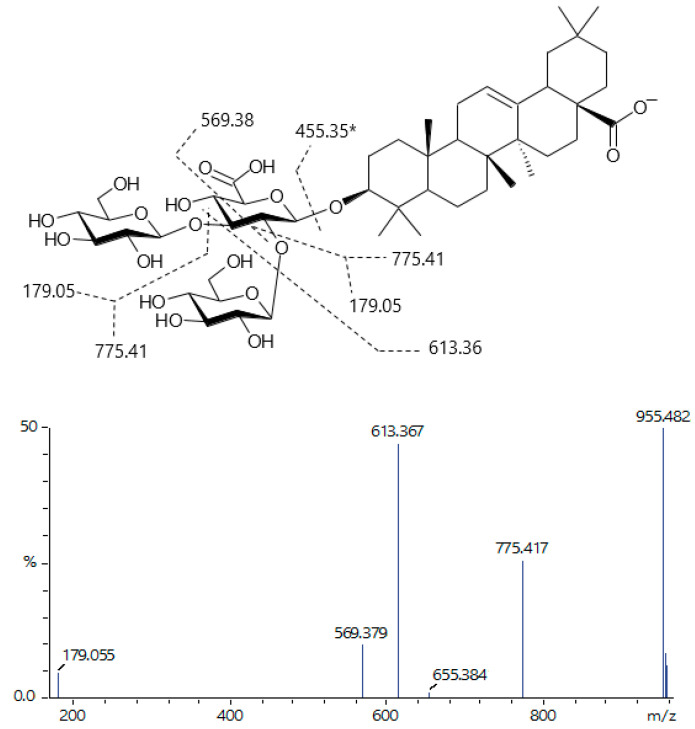
A saponin found in *P. grandifolia*, depicted based on the structure reported by Sahu et al. [41], and its mass spectrum assignment. Other saponins that share a similar structural core, composed of oleanolic acid attached by a glucuronic acid. The glycan chains are elongated with glucose units up to three units. Due to several isomers found, other glycosidic linkages, and another aglycone, such as ursolic acid, must be regarded as necessary to explain this variability. * The aglycone fragment was not produced from all compounds.

Three isomers (**87**, **93**, **94**) were found at *m*/*z* 955.482 [M-H]^−^, identified as from a triglycosyl saponin (Figure 6). As in compound **85**, losses of 162/180 Da indicated the loss of hexoses, and the common fragment at *m*/*z* 613.368 confirmed the presence of the uronic acid residue linked to the aglycone. Isomers can be derived from aglycone isomers (e.g., ursolic/oleanolic acid), monosaccharide epimers (e.g., galactose and glucose, or galacturonic and glucuronic acid), or different linkage sites. Only a single structure of these isomers was previously described [41].

Compounds **88**, **90**, **96**, **98,** and **99** were all found at *m*/*z* 793.430 [M-H]^−^ with similar fragments, at *m*/*z* 631.383 and 613.368 (loss of a hexose), confirming the uronic acid linked to aglycone, and these isomers were characterized as diglycosyl saponins (Table 2). The compound **100**, at *m*/*z* 835.441, with fragments at *m*/*z* 655.380 and 611.388, could arise from an acetylation version of compounds **88**, **90**, **96**, **98**, and **99**. The presence of two saponin monoglycosides (**102**, **103**), which were found at *m*/*z* 631.379, yielding fragments at *m*/*z* 613.370, confirming the dehydration tendency, and *m*/*z* 455.350, from the intact aglycone, consistent with the stated ursolic/oleanolic acids, confirmed their linkage with uronic acid.

Other potential saponins composed of different monosaccharides or aglycones were also found. Compound **84**, at *m*/*z* 985.492 [M-H]^−^, was 30 Da (CH_2_O), higher than saponins **87**, **93**, **94** (*m*/*z* 955.481). The fragments at *m*/*z* 805.430 (−180) indicated the loss of a hexose unit, followed by *m*/*z* 643.379 (−162) from loss of another hexose dehydrated residue. This fragment retained the additional 30 Da higher than the usual fragment observed for other saponins (*m*/*z* 613.368), suggesting the presence of a different aglycone (C_31_H_50_O_4_), such as hederagenin methyl ester.

Compound **86**, at *m*/*z* 1087.523 [M-H], was 30 Da lower than compound **80**, and compound **95**, at *m*/*z* 925.471 [M-H], was 30 Da lower than compounds **87**, **93**, and **94** (*m*/*z* 955.481). These compounds could result from replacing a hexose unit with a pentose one; nevertheless, they did not yield sufficient fragments to confirm their structures.

The compound **83**, *m*/*z* 711.374 [M+HCOO]^−^, gave fragments at *m*/*z* 665.370 from the loss of adduct, along with *m*/*z* 503.317 and 179.053, confirming the presence of a hexose. The probable aglycone was found forming the deprotonated ion at *m*/*z* 503.318 (**89**). Although the mass error was higher than expected, the aglycone of this saponin could be madecassic acid (expected *m*/*z* 503.337).

Compound **92**, *m*/*z* 809.435, and fragments at *m*/*z* 629.363 (loss of a hexose) and *m*/*z* 585.384 could indicate the presence of an additional hydroxyl group in aglycone, such as hydroxyursolic acid (hederagenin), which perfectly matches the fragments observed.

The compound **97** appeared at *m*/*z* 791.415 [M-H]^−^ with a main fragment at *m*/*z* 611.353. These ions were 2 Da smaller than compounds **88**, **90**, **96**, **98**, and **99** (*m*/*z* 793.430), consistent with an additional unsaturation in the aglycone, namely dehydro-ursolic acid, which was previously characterized in saponins from other plants [42].

## 3. Discussion

This work represents the first comprehensive characterization of phytochemicals in *Pereskia aculeata* and *P. grandifolia* using two complementary chromatographic methodologies, GC-MS and LC-MS. This combination provides a robust approach that overcomes common limitations of LC-MS, particularly for complex phytochemical samples, which often contain numerous isomers that are not resolved by mass spectrometric analysis. While LC-MS provides primary information on overall composition, GC-MS was critical for identifying monosaccharides from the glycosides and fatty acids in lipids. Such MS investigations with this level of detail are becoming scarce, as most studies are merely descriptive and do not thoroughly examine the MS profile to identify molecular fingerprints.

Although *P. aculeata* has been previously investigated and reported as rich in phenolic compounds, notably flavonol glycosides and phenolic acids [3,4], phytochemical data on *P. grandifolia* remained scarce. By applying these advanced analytical techniques, our study not only confirms and expands previous findings on *P. aculeata* but also uncovers, for the first time, the presence of a diverse range of saponins in *P. grandifolia*. This comparative analysis offers novel insights into the chemical basis underlying the distinct biological activities exhibited by these species. The marked differences in chemical composition between the two species likely reflect intrinsic biosynthetic strategies. *P. aculeata* accumulates phenolic compounds, particularly a greater variety of glycosylated flavonoids, typically associated with photoprotection and defense against oxidative stress. In contrast, *P. grandifolia* showed two abundant flavonoids; this species seems to invest more energy into the synthesis of triterpenoid saponins, structurally diverse metabolites widely recognized for their ecological roles in pathogen defense and herbivory control.

The stronger chemical antioxidant capacity observed in *P. aculeata* correlates with its higher content of flavonoids and phenolic acids, falling within the same range as those reported for other flavonoid-rich extracts, reinforcing the central role of these compounds as radical scavengers, metal chelators, and redox modulators, beyond several different properties, as anti-inflammatory, vasoprotective, and immunomodulatory effects [43,44,45,46,47,48,49]. By contrast, *P. grandifolia*, with a markedly lower phenolic content, displayed weaker chemical antioxidant responses, which is consistent with previous findings on extracts poor in polyphenols.

Interestingly, despite its lower phenolic abundance, the detection of multiple saponins in *P. grandifolia* suggests complementary antioxidant mechanisms, likely indirect, through modulation of endogenous enzymatic defenses such as superoxide dismutase, catalase, and glutathione peroxidase. This may be the key reason for the higher cellular antioxidant activity observed in the butanolic fraction of this species, which achieved ROS inhibition above 90%. In contrast, PA-But achieved only 45% inhibition, as the best result. Indeed, saponins from other plant sources were investigated for their effects on cellular oxidative stress, indicating that the primary mechanisms involve upregulating antioxidant enzymes, such as CAT, NRF2, and GCL, as well as restoring glutathione homeostasis [50]. Therefore, it could be the primary pathway underlying the anti-ROS effect observed after incubation with PG-But, which may also explain the lower effects of PA-But.

The pharmacological potential of saponins identified in *P. grandifolia* deserves particular attention. Beyond their indirect antioxidant effects, saponins are widely reported to have diverse biological activities, including anti-inflammatory effects through downregulation of mediators such as TNF-α, IL-6, and COX-2, cytotoxicity against tumor cells via apoptosis induction, and antimicrobial activity against bacteria, fungi, and protozoa [50,51,52]. Such findings position *P. grandifolia* as a promising source of bioactive metabolites distinct from those found in *P. aculeata*. Given the growing interest in plant-based superfoods and functional ingredients, both species represent valuable candidates not only as nutrient-rich vegetables but also as potential raw materials for nutraceutical and pharmaceutical applications.

Regardless of the significant advances in chemical composition and mass spectrometry profiles presented in this work, along with the distinct antioxidant effects observed in both plant components, more advanced assays are needed to fully understand the potential of these species. These assays could include investigating the cellular antioxidant pathway to better understand the observed differences between species, beyond differences in chemical composition. However, challenges in analyzing complex mixtures hinder compound identification, owing to the limited availability of reference standards and the inefficiency of nuclear magnetic resonance (NMR) analysis for such mixtures. This also impacts biological investigations, as different compounds may act synergistically or exhibit opposing effects, leading to an over- or underestimation of the pharmacological contribution of specific metabolites. This could be a reasonable explanation for the low cellular antioxidant activity observed in the PA-But fraction. One way to overcome these difficulties is to implement additional fractionation and purification protocols, which are laborious and beyond the scope of this work, but underscore the importance of future studies that involve pure compounds, large-scale metabolite validation, and in vivo assays to confirm the pharmacological effects suggested by our data.

Despite limitations, our results provide new and complementary insights into the phytochemical and biological diversity of *Pereskia* species. The dual antioxidant profile observed in this study, in which *P. aculeata*, rich in phenolic compounds and showing greater chemical antioxidant activity, was compared with *P. grandifolia*, which was rich in saponins and exhibited stronger cellular antioxidant activity, highlights the complexity and versatility of this genus as a source of bioactive compounds. Furthermore, this study establishes the first comprehensive characterization of *Pereskia* species by GC-MS and LC-MS, laying the groundwork for future investigations into their nutraceutical value and potential therapeutic applications.

## 4. Materials and Methods

### 4.1. Botanical Material

Leaves from *P. aculeata* and *P. grandifolia* were provided and identified by Dr. Ezilda Jacomassi, from the Botanical Garden of the Universidade Paranaense (Umuarama, Paraná State, Brazil) at 430 meters above sea level (S23°46′11.3″–W53°16′412″), where these species are cultivated. Leaf samples of these species were collected in August/September 2023. The voucher specimens were deposited at the Botanical Garden Herbarium of the Universidade Paranaense under exsiccate HHMUP-88 (*Pereskia aculeata* Mill.) and HHMUP-142 (*Pereskia grandifolia* Haw.). Both species were registered in the National System for the Management of Genetic Heritage and Associated Traditional Knowledge under registration numbers A578420, A464E1A, and A5E5EFC from UNIPAR and AAA483B from IPPPP.

### 4.2. Chemicals

All chemicals and reagents were purchased from Sigma-Aldrich (St. Louis, MO, USA), unless otherwise specified. Formic acid (98%) and acetonitrile were LC-MS-grade, and ethanol, methanol, butanol, ethyl acetate, propanol, chloroform, *n*-hexane, and acetic acid were HPLC-grade. Other solvents and reagents were P.A. grade. Water was type 1 ultrapure (Milli-Q, Merck Millipore, Molsheim, France).

### 4.3. Leaf Extraction and Liquid/Liquid Partition

The leaves of *P. aculeata* and *P. grandifolia* were ground in a blender, and 100 g of each was extracted with 70% ethanol at 60 °C for 2 h, with the extraction repeated three times. The extracts were combined and evaporated under reduced pressure. A 1 g portion of each extract was dissolved in water (200 mL) and then fractionated with chloroform (200 mL). The organic layer was recovered, and this process was repeated three times. The chloroform layers were combined and evaporated under reduced pressure, yielding the fractions PA-Chl (from *P. aculeata*) and PG-Chl (from *P. grandifolia*). The aqueous layer was subjected to a second fractionation with a mixture of *n*-butanol and ethyl acetate (8:2, *v*/*v*). After three partition cycles, the organic layers were combined and evaporated under reduced pressure, yielding PA-But from *P. aculeata* and PG-But from *P. grandifolia,* respectively. The residual aqueous layers were freeze-dried at −50 °C (Liobras, São Carlos, Brazil), giving the fractions PA-Aq (*P. aculeata*) and PG-Aq (*P. grandifolia*). These fractions were stored at −20 °C until analysis and assays were performed.

### 4.4. DPPH Free Radical Scavenging Assay

The experiment followed the procedure described by [53] with adaptations. Fractions PA-But, PA-Aq, PG-But, and PG-Aq were diluted in ethanol to obtain concentrations ranging from 1000 to 1.9 μg·mL^−1^. The obtained solutions (143 μL) were subsequently mixed with 57 μL of an ethanolic DPPH solution (0.3 mM). The mixture was incubated for 30 min and protected from light. Absorbance was determined using a spectrophotometer (UV-VIS 1601, Shimadzu, Kyoto, Japan) at 540 nm. The negative control was performed using a DPPH solution and ethanol. The blank was formed by mixing ethanol (57 μL) with each of the obtained solutions, ensuring a specific blank for each concentration. The samples were diluted to their respective IC_%,_ resulting in different concentrations. The results were compared with a standard of ascorbic acid. The assay for each concentration per sample was conducted in triplicate under the same analysis conditions, and the averages were used to calculate the DPPH inhibition percentage relative to the negative control using the following formula:

%Inhibition DPPH = 100 − [(Abs_sample_ − Abs_blank_)/Abs_control_] × 100

A graph of the DPPH inhibition percentage against concentration was obtained. The IC_50_ of the analyzed sample was determined from the linear regression equation. The antioxidant activity of the extracts was classified according to the following criteria: IC_50_ < 50 μg·mL^−1^ = very strong, 50–100 μg·mL^−1^ = strong, 101–250 μg·mL^−1^ = moderate, 251–500 μg·mL^−1^ = weak, and >500 μg·mL^−1^ = inactive [54].

### 4.5. Evaluation of Activity Through the Formation of the Phosphomolybdenum Complex

The assay was conducted following the method described by [55]. Fractions PA-But, PA-Aq, PG-But, and PG-Aq were dissolved in ethanol (0.3 mL; 200 μg·mL^−1^) and added to a tube containing 1 mL of the phosphomolybdenum complex solution and 1.5 mL of distilled water, and then incubated at 90 °C for 90 min. After the reaction time, the absorbance was measured at 690 nm using a Multiskan FC microplate reader (Thermo Scientific, Waltham, MA, USA). The absorbances of the samples were compared with the absorbance of the ascorbic acid standard at the same concentrations. The results were expressed as relative antioxidant activity (RAA%, compared to ascorbic acid), according to the following equation:

RAA% (ascorbic acid) = [(Abs_sample_ − Abs_blank_)/(Abs_ascorbic acid_ − Abs_blank_)] × 100

### 4.6. Determination of Total Flavonoids

Total flavonoids were determined by the method described by [56] in triplicate, using a standard curve of quercetin dissolved in ethanol at concentrations ranging from 10 to 500 μg·mL^−1^. First, 100 µL of each fraction (500 μg·mL) or 100 µL of the quercetin standard was added to a 96-well plate. Then, 100 µL of AlCl_3_ in methanol (2% *w*/*v*) was added to each well, incubated for 60 min, and protected from light. Analysis was performed on a Multiskan FC microplate reader (ThermoScientific, Waltham, MA, USA) at 414 nm against a blank. Quantification was carried out using the external calibration curve method, with quercetin as the reference standard. The linear regression equation obtained (y = 0.009x − 0.0136; R^2^ = 0.9976) was applied to interpolate the absorbance values of the samples and calculate their respective concentrations, which were then expressed as mg of quercetin equivalents per gram of extract (mg EQ/g).

### 4.7. Determination of Total Polyphenol Content

The total polyphenol was determined in triplicate by the Folin-Ciocalteu method [57]. Folin-Ciocalteu reagent (100 µL) diluted in water (10%, *v*/*v*), 20 µL fractions at 500 μg·mL^−1^ or gallic acid ranging from 10 to 500 μg·mL^−1^, and 80 µL of sodium carbonate solution (7.5%, *w*/*v*) were added in a 96-well plate, incubated for 60 min, protected from light. The analysis was performed in a Multiskan FC microplate reader (Thermo Scientific, Waltham, MA, USA) at 690 nm. Quantification was performed using the external calibration curve method, with gallic acid as the reference standard, prepared in ethanol. The linear regression equation obtained (y = 0.0024x + 0.0468; R^2^ = 0.9927) was applied to interpolate the absorbance values of the samples and calculate their respective concentrations, which were then expressed as milligrams of gallic acid equivalents per gram of extract (mg GAE/g).

### 4.8. Cellular Antioxidant Activity Assay

The cellular antioxidant activity (CAA) was assessed by measuring the ability of the plant fractions to inhibit reactive oxygen species (ROS) production in response to an external stimulus. CACO-2 cells were cultured in DMEM-F12 medium, supplemented with 10% bovine fetal serum and 1% penicillin and streptomycin, and seeded into 96-well microplates at a density of 5 × 10^5^ cells/well and held for 24 h at 37 °C and 5% CO^2^, to ensure cellular adherence and establish a confluent monolayer. To access the intracellular antioxidant activity, cells were co-incubated with the fluorescent probe 2′,7′-dichlorofluorescein diacetate (DCFH-DA) at a final concentration of 20 μM, and with the PA-But and PG-But fractions, at concentrations of 50, 100, 300, 600, and 1000 μg·mL^−1^, for 60 min at 37 °C, following a modified version of the protocol previously described [58]. This incubation period enabled intracellular deacetylation of DCFH-DA to 2′,7′-dichlorofluorescein (DCFH). After this incubation period, the wells were washed with phosphate-buffered saline (PBS) to remove any non-internalized DCFH-DA and samples, the cells were then incubated with the ROS stimulator, 5-fluorouracil (5-FU), at 25 mg·mL^−1^. Fluorescence intensity was immediately monitored for 60 min, with readings taken every 5 min, with excitation/emission of 500 nm/529 nm, using a Varioskan LUX Microplate Reader (Thermo-Scientific—Waltham, MA, USA). Data acquisition was performed in SkanIt RE v. 7.1 (Thermo-Scientific—Waltham, MA USA) and analysis in GraphPad Prism 10 (San Diego, CA, USA), which provided the area under the curve (AUC), used to calculate the total CAA, as follows:

CAA Units = 100 − (AUC_Antioxidant_/AUC_Control_) × 100

### 4.9. Monosaccharide and Aglycone Analysis

To remove residual free mono- or oligosaccharides, 5 mg of the fractions PA-But and PG-But were dissolved in 2 mL ultrapure water and partitioned with 2 mL butanol/ethyl acetate (2:1 *v*/*v*) three times; the aqueous phase was discarded and the organic layers were analyzed by thin layer chromatography (TLC) to monitor the presence of free sugars, using silica gel 60G (Sigma-Aldrich St. Louis, MO, USA), developed with EtOAc:H_2_O:HOAc:HCO_2_H (9:2.3:1:1, *v*/*v*) and stained with orcinol–H_2_SO_4_ at 100 °C [59]. After confirming their absence, the samples were hydrolyzed in 1 M TFA, for 4 h at 100 °C. The hydrolysate was partitioned with ethyl acetate/water (1:1, *v*/*v*), and the organic layer was evaporated under a nitrogen stream and reserved for further analysis of the aglycone. The aqueous phase was evaporated under a N_2_ stream, and the monosaccharides were reduced with NaBH_4_ and acetylated with pyridine/acetic anhydride (1:1, *v*/*v*) as previously [26,59]. The resulting alditol acetates were analyzed by gas chromatography coupled with mass spectrometry (GC-MS).

### 4.10. Fatty Acid Analysis

Fatty acids from the fractions PA-But and PG-But (1 mg each) were converted to their methyl ester derivatives by dissolving them in 500 μL of 1 M methanol-HCl and heating at 80 °C for 1 h. The resulting fatty acid methyl ester (FAME) was extracted by adding *n*-hexane (1 mL) and 500 μL of water. The upper layer was recovered, and the FAMEs were analyzed by GC-MS [60].

### 4.11. Gas Chromatography Coupled to Mass Spectrometry Analysis

Alditol acetates and FAME derivatives were analyzed by GC-MS QP2020 (Shimadzu, Kyoto, Japan), coupled to a single quadrupole mass spectrometer equipped with an AOC-6000 autosampler. The column was a 30-m SH-RTX-5MS (Shimadzu, Kyoto, Japan) fused-silica capillary column with an inner diameter of 0.25 mm and a film thickness of 0.25 μm. The carrier gas was helium (analytical grade, 5.0), with the pressure controlled at 90.7 kPa (flow rate of 1.54 mL·min^−1^).

The chromatography was developed with an initial column temperature of 50 °C, then heated to 150 °C at a rate of 20 °C·min^−1^, then to 200 °C at 5 °C·min^−1^ held for 5 min, and to 250 °C at 25 °C·min^−1^ held for 5 min, with a total running time of 28 min. Other GC-MS components were held at 250 °C (injector, ionization source, and MS interface). Samples were dissolved in acetone with 1 μL of injection, in split mode of 1:10. Data were collected in Scan Mode/Total Ion Current (TIC), ranging from *m*/*z* 50–500, with MS operating at electron ionization (EI) mode, with 70 eV. In comparison with authentic standards, the monosaccharides (as alditol acetates) and fatty acids (as FAMEs) were identified by their relative retention times and EI-MS profiles. Data acquisition and analysis were performed with GCMSsolutions v. 4.53SP1 (Shimadzu, Kyoto, Japan).

### 4.12. Liquid Chromatography—Mass Spectrometry Analysis

The phytochemical composition of PA-But and PG-But was analyzed using an LC-MS-9050 system (Shimadzu, Kyoto, Japan), equipped with a binary high-pressure pump (10,000 psi), autosampler, and column oven. Detection was performed using high-resolution mass spectrometry with a Quadrupole-Time-of-Flight (Q-ToF) mass analyzer and an atmospheric-pressure ionization (API) electrospray ionization (ESI) source. Chromatographic separation was achieved on a reversed-phase C18 column (Titan—Supelco, Bellefonte, PA, USA), 100 × 2.1 mm, 1.9 µm particle size, maintained at 40 °C. The mobile phase consisted of ultra-pure water and acetonitrile containing 0.1% formic acid. A gradient elution was applied at a flow rate of 350 µL·min^−1^, starting with 5% acetonitrile (held for 1 min), increasing to 80% over 20 min, then to 100% at 22 min, held until 26 min, followed by return to initial conditions at 30 min, and held for a re-equilibration time of 5 min. Samples were prepared at a concentration of 1 mg·mL^−1^ in a methanol/water solution (1:1, *v*/*v*), filtered through a 0.22 µm × 13 mm PTFE syringe filter (Merck, Darmstadt, Germany), and 2 µL were injected for analysis.

Mass spectrometry detection was performed in both positive and negative ion modes, with a scan time of 0.1–30 min and an MS range of *m*/*z* 100–2000 at an event time of 0.3 s. The ion source temperatures were as follows: interface at 300 °C, heat block at 400 °C, desolvation line at 250 °C, and desolvation temperature at 526 °C. The gas flow rates were set to 3.0 L·min^−1^ (nebulizing gas), 10 L·min^−1^ (heating gas), and 10 L·min^−1^ (drying gas), with a capillary voltage of 2.5 kV.

For MS^2^ analysis, the mass spectrometer operated in data-dependent acquisition (DDA) mode, with 5 events and a scan mass range of *m*/*z* 100–1500. Precursor ion isolation was performed with a unit Q1 resolution and a threshold intensity of 2000, at charge states of 1 and 2, and an event time of 0.1 s. The fragments were obtained by collision-induced dissociation (CID) at 30 eV (±17 eV spread), using argon at 270 kPa as the collision gas. Data were processed using LabSolutions v. 5.120 and LabSolutions Insight Explorer CSD v. 1.00 software (Shimadzu, Kyoto, Japan), and spectral comparisons were performed using the Pubchem (https://pubchem.ncbi.nlm.nih.gov), MoNa (https://mona.fiehnlab.ucdavis.edu), and Lotus (https://lotus.naturalproducts.net) databases, accessed between April and May 2025, and literature reports. In addition, authentic standards of rutin, quercetin, kaempferol, urosolic acid, and oleanolic acid were used to support compound identification.

### 4.13. Statistical Analysis

The chemical antioxidant assays were performed in triplicate, and the results are expressed as the mean ± standard error. The cellular antioxidant assays were performed in quadruplicate, repeated on three independent days. One-way analysis of variance (ANOVA) followed by Tukey’s multiple comparisons test was used to determine significance, using GraphPad Prism 10 (GraphPad Software, San Diego, CA, USA). Statistical significance was considered at *p* < 0.05.

## 5. Conclusions

This work provides a solid chemical and biological basis for understanding the bioactive potential of *Pereskia* species, supporting their valorization as underexplored plant resources with relevant nutritional and pharmacological properties, by offering the first comprehensive comparison of *P. aculeata* and *P. grandifolia* leaves, integrating phytochemical analysis by mass spectrometry and antioxidant evaluation. The results demonstrate that *P. aculeata* has higher levels of total phenolics and flavonoids, which are directly reflected in its superior chemical antioxidant capacity. In contrast, *P. grandifolia* exhibited a distinct phytochemical profile, notably characterized by higher saponin content. This species showed greater cellular antioxidant activity, reducing ROS levels more effectively than *P. aculeata*, despite its lower phenolic/flavonoid content. The fact that *P. aculeata* compounds were less effective in reducing ROS levels is unclear, but may result from the presence of stimulatory compounds that act in an antagonistic manner to the phenolics/flavonoids present in the PA-But fraction. These findings highlight the chemical diversity within the genus *Pereskia* and reinforce the nutritional and pharmacological relevance of these species, which can be considered promising sources of bioactive compounds for the development of functional foods, dietary supplements, and nutraceuticals. Future studies should focus on isolating the structurally elucidated metabolites, evaluating their individual biological activities, and exploring synergistic and antagonistic effects among these plant components.

## Figures and Tables

**Table 1:** Assessment of antioxidant potential, total phenolics, and flavonoids.

Sample	DPPH (IC_50_—μg·mL^−1^) ^1^	Phosphomolybdenum Complex (%RAA) ^2^	Total Polyphenols ^3^	Total Flavonoids ^4^
PA-But	22.1 ± 5.9 ^a^	43.1 ± 1.5 ^a^	102 ± 3 ^a^	69.1 ± 0.5 ^a^
PA-Aq	60 ± 3.1 ^b^	27.8 ± 1.5 ^b^	62.2 ± 0.2 ^b^	31.5 ± 0.5 ^b^
PG-But	53.3 ± 0.6 ^b^	22.1 ± 1.8 ^c^	28.8 ± 0.4 ^c^	40.8 ± 2.1 ^c^
PG-Aq	86.8 ± 5.8 ^c^	10.8 ± 1.6 ^d^	17.2 ± 0.2 ^d^	25 ± 2.3 ^d^
Ascorbic acid	6.1 ± 0.5 ^d^	100 *	-	-

Values in each column that have a distinct letter were statistically different (*p* < 0.05), while the same letter indicated the lack of differences (*p* > 0.05), as measured by one-way analysis of variance (ANOVA) followed by Tukey’s multiple comparison test. ^1^ IC_50_: concentration of samples required to inhibit the formation of DPPH radicals by 50%. ^2^ The results were expressed as percentages, with ascorbic acid * serving as the 100% standard. ^3^ Quantification of total polyphenols as mg of gallic acid equivalent per g of extract, and ^4^ quantification of total flavonoids as mg of quercetin equivalent per g of extract.

**Table 2:** Phytochemical profile from the fractions PA-But (*P. aculeata*) and PG-But (*P. grandifolia*), tentatively identified based on mass spectra data.

Peak	*t*_R_	Precursor Ion	Fragments	Molecular Formula	Tentatively Identification
**PA-But**					
1	0.835	377.08402	341.107, 215.033, 179.054	C_12_H_22_O_11_ [M+Cl]^−^	Sucrose
2	1.333	191.01834	129.018, 111.007, 87.007, 85.028	C_6_H_8_O_7_ [M-H]^−^	Citric acid
3	1.919	176.07080	161.047, 160.039, 149.061, 134.023		n.i.
4	2.095	197.00800	153.018, 151.002, 125.023, 109.028, 107.013, 83.130, 65.002	C_8_H_8_O_6_ [M-H]^−^	Dihydroxybenzene-dicarboxylic acid
5	2.870	315.10704	153.054, 123.044	C_14_H_20_O_8_ [M-H]^−^	Hydroxytyrosol glucoside
6	3.273	153.01835	123.044, 109.028, 108.020, 91.018, 81.033	C_7_H_6_O_4_ [M-H]^−^	Protocatechuic acid
7	3.922	341.08578	179.033, 135.044	C_15_H_18_O_9_ [M-H]^−^	Caffeoyl glucoside
8	4.892	175.06023	115.039, 113.059, 85.065	C_7_H_12_O_5_ [M-H]^−^	Isopropylmalic acid
9	5.069	291.09686	142.065, 119.049		n.i.
10	5.121	526.18929	218.080		n.i.
11	5.209	487.14361	179.034, 135.045	C_21_H_28_O_13_ [M-H]^−^	Caffeoyl rutinoside
12	5.395	341.08622	179.033, 135,044	C_15_H_18_O_9_ [M-H]^−^	Caffeoyl glucoside
13	5.513	325.09098	163.038, 119.049	C_15_H_18_O_8_ [M-H]^−^	*p*-Coumaroyl glucoside
14	5.734	218.08103	188.070, 162.055, 135.044, 82.029	C_12_H_13_NO_3_ [M-H]^−^	n.i.
15	6.011	315.10672	153.054	C_14_H_20_O_8_ [M-H]^−^	Hydroxytyrosol glucoside isomer
16	6.262	179.03401	135.044	C_9_H_8_O_4_ [M-H]^−^	Caffeic acid
17	6.386	333.02271	197.008, 178.997, 153.017, 151.002	C_15_H_10_O_9_ [M-H]^−^	Protocatechuic-dihydroxybenzene-dicarboxylic acid.
18	6.613	447.14793	401.145, 269.098, 161.043	C_25_H_22_O_5_ [M+HCOO]^−^	n.i.
19	7.162	435.22101	n.d.	C_27_H_32_O_5_ [M-H]^−^	n.i.
20	7.232	567.26187	521.257, 389.217, 293,085, 161.045	C_24_H_42_O_12_ [M+HCOO]^−^	Alangionoside B isomer
21	7.701	295.04444	179.033, 135.044, 133.013, 115.002	C_13_H_12_O_8_ [M-H]^−^	Caffeoyl malic acid
22	7.897	359.09657	151.038	C_14_H_18_O_8_ [M+HCOO]^−^	Methoxybenzoyl hexoside
23	8.070	371.09643	249.060, 231.050, 121.028	C_16_H_20_O_10_ [M-H]^−^	Benzoyl glycoside
24	8.625	163.03886	119.049, 93.033	C_9_H_8_O_3_ [M-H]^−^	*p*-Coumaric acid
25	8.927	755.19918	609.143, 300.024	C_33_H_40_O_20_ [M-H]^−^	Quercetin (Rha)_2_-hexoside
26	9.040	755.19850	609.142, 301.030	C_33_H_40_O_20_ [M-H]^−^	Quercetin (Rha)_2_-hexoside
27	9.365	741.18404	609.140, 300.025	C_32_H_38_O_20_ [M-H]^−^	Quercetin Ara-Rha-hexoside
28	9.559	741.18352	609.140, 300.025	C_32_H_38_O_20_ [M-H]^−^	Quercetin Ara-Rha-hexoside
29	9.859	739.20474	284.031	C_33_H_40_O_19_ [M-H]^−^	Kaempferol (Rha)_2_-hexoside
30	9.995	595.12665	300.025	C_26_H_28_O_16_ [M-H]^−^	Quercetin Ara-hexoside
31	10.157	595.12705	463.086, 300.025	C_26_H_28_O_16_ [M-H]^−^	Quercetin Ara-hexoside
32	10.255	609.14257	301.033, 300.025	C_27_H_30_O_16_ [M-H]^−^	Rutin (isomer)
33	10.375	725.18896	593.142, 285.038, 284.030	C_32_H_38_O_19_ [M-H]^−^	Kaempferol Ara-Rha-hexoside
34	10.633	725.18895	593.143, 285.038, 284.030	C_32_H_38_O_19_ [M-H]^−^	Kaempferol Ara-Rha-hexoside
35	10.695	463.08488	301.033, 300.025	C_21_H_20_O_12_ [M-H]^−^	Quercetin hexoside
36	10.862	755.19913	623.157, 315.049, 314.041, 299.016	C_33_H_40_O_20_ [M-H]^−^	Isorhamnetin Ara-Rha-hexoside
37	10.981	463.08520	301.033, 300.025	C_21_H_20_O_12_ [M-H]^−^	Quercetin hexoside
38	11.123	579.13170	447.092, 285.038, 284.035	C_26_H_28_O_15_ [M-H]^−^	Kaempferol Ara-hexoside
39	11.287	593.14714	285.037, 284.035	C_27_H_30_O_15_ [M-H]^−^	Kaempferol Rha-hexoside
40	11.469	262.07029	218.080, 200.069, 146.060, 115.002	C_13_H_13_NO_5_ [M-H]^−^	Isoquinoline alkaloid
41	11.586	609.14161	315.049, 314.042	C_27_H_30_O_16_ [M-H]^−^	Isorhamnetin Ara-hexoside
42	11.877	447.09039	285.038, 284.035	C_21_H_20_O_11_ [M-H]^−^	Kaempferol hexoside
43	12.025	623.15751	315.049, 314.041	C_28_H_32_O_16_ [M-H]^−^	Isorhamnetin Rha-hexoside
44	12.514	477.10041	315.048, 314.042	C_22_H_22_O_12_ [M-H]^−^	Isorhamnetin hexoside
45	12.802	477.10011	314.041	C_22_H_22_O_12_ [M-H]^−^	Isorhamnetin hexoside
46	13.133	579.13207	315/315	C_26_H_28_O_15_ [M-H]^−^	Isorhamnetin Ara-arabinoside
47	13.134	503.11619	323.074, 179.033, 135.044	C_24_H_24_O_12_ [M-H]^−^	Dicaffeoyl-hexose
48	16.560	301.03295	178.997, 151.002, 121.028, 107.013	C_15_H_10_O_7_ [M-H]^−^	Quercetin
49	19.611	327.21548	229.143, 211.133, 171.101	C_18_H_32_O_5_ [M-H]^−^	Trihydroxy-octadecadienoic acid
50	19.716	327.21516	291.195, 229.143, 211.132, 171.100	C_18_H_32_O_5_ [M-H]^−^	Trihydroxy-octadecadienoic acid
51	19.868	327.21532	229.144, 211.132, 171.100	C_18_H_32_O_5_ [M-H]^−^	Trihydroxy-octadecadienoic acid
52	20.421	955.48309	775.422, 613.370, 569.381, 179.054	C_48_H_76_O_19_ [M-H]^−^	Triglycosyl-saponin
53	22.506	793.43180	613.369, 569.378	C_42_H_66_O_14_ [M-H]^−^	Diglycosyl-saponin
54	22.660	721.35951	675.352, 415.142, 397.132, 277.216	C_33_H_56_O_14_ [M+HCOO]^−^	Digalactosylmonoacylglycerol-C_18:3_
55	22.779	593.26897	277.216, 241.009, 152.994	C_27_H_47_O_12_P [M-H]^−^	lyso-Phosphatidylinositol-C_18:3_
56	22.871	721.35988	675.355, 415.142, 397.132, 277.215, 235.081	C_33_H_56_O_14_ [M+HCOO]^−^	Digalactosyl-monoacylglycerol-C_18:3_
57	23.027	593.26906	413.209, 315.046, 277.215, 241.009, 152.994	C_27_H_47_O_12_P [M-H]^−^	lyso-Phosphatidylinositol-C_18:3_
58	23.770	595.28406	315.044, 279.231, 241.011, 152.994	C_27_H_49_O_12_P [M-H]^−^	lyso-Phosphatidylinositol-C_18:2_
59	23.813	699.37540	653.370, 415.142, 397.131, 305.084, 287.076, 255.231, 179.053	C_31_H_58_O_14_ [M+HCOO]^−^	Digalactosylmonoacylglycerol-C_16:0_
60	23.886	265.14582	96.959, 79.956	C_12_H_26_O_4_S [M-H]^−^	SDS
61	24.231	571.28434	315.045, 255.231, 241.009, 152.994	C_25_H_49_O_12_P [M-H]^−^	lyso-phosphatidylinositol-C_16:0_
62	24.988	555.28003	299.041, 255.230, 225.005, 164.985, 125.025	C_25_H_48_O_11_S [M-H]^−^	Sufoquinovosylmonoacylglycerol-C_16:0_
63	0.900	180.10012	163.073, 145.062, 117.068, 115.052, 91.052	C_10_H_13_NO_2_ [M+H]^+^	Solsolinol isomer 1
64	1.336	180.10017	163.073, 145.062, 117.068, 115.052, 91.052	C_10_H_13_NO_2_ [M+H]^+^	Solsolinol isomer 2
65	2.305	166.08455	120.079, 103.052, 91.052, 77.037	C_9_H_11_NO_2_ [M+H]^+^	Phenylalanine
66	3.922	205.09504	188.068, 146.057, 118.063, 115.052, 91.052	C_11_H_12_N_2_O_2_ [M+H]^+^	Tryptophan
67	4.113	219.11031	188.068, 146.058, 132.078, 118.063, 91.052	C_12_H_14_N_2_O_2_ [M+H]^+^	Abrine
68	5.086	247.14134	188.067, 170.057, 146.057, 118.062, 91.051, 60.078	C_14_H_19_N_2_O_2_ [M]^+^	Hipaphorine
69	22.352	318.29637	300.285, 282.274, 270.264, 264.264, 252.262	C_18_H_39_NO_3_ [M+H]^+^	Phytosphingosine
70	23.997	496.33387	478.320, 184.069, 124.996, 104.103	C_24_H_51_NO_7_P [M]^+^	lyso-Phosphatidylcholine-C16
**PG-But**					
71	5.502	527.22978	481.223, 349.186, 149.042	C_21_H_38_O_12_ [M+HCOO]^−^	Distyloside A pentoside
72	5.657	395.18859	349.185, 179.055	C_16_H_30_O_8_ [M+HCOO]^−^	Distyloside A
73	5.968	527.22951	481.224	C_21_H_38_O_12_ [M+HCOO]^−^	Distyloside A pentoside
74	6.052	278.06458	216.062, 162.054, 132.028, 119.049	C_13_H_13_NO_6_ [M-H]^−^	Isoquinoline alkaloid
75	6.607	447.14683	401.142, 269.100, 193.048, 161.043	C_18_H_26_O_10_ [M+HCOO]^−^	Benzyl primeveroside
76	7.271	431.18869	385.183, 223.132, 205.121, 161.044, 153.090	C_19_H_30_O_8_ [M+HCOO]^−^	Roseoside isomer
23	8.079	371.09519	249.059, 121.027	C_16_H_20_O_10_ [M-H]^−^	Benzoyl glycoside
25	8.927	755.19844	609.141, 301.032, 300.024	C_33_H_40_O_20_ [M-H]^−^	Quercetin (Rha)_2_-hexoside
29	9.864	739.20305	593.148, 284.029/285.037	C_33_H_40_O_19_ [M-H]^−^	Kaempferol (Rha)_2_-hexoside
32	10.267	609.14050	301.033, 300.024	C_27_H_30_O_16_ [M-H]^−^	Rutin (isomer)
77	11.478	262.07073	218.080, 200.070, 146.059, 115.002	C_13_H_13_NO_5_ [M-H]^−^	Isoquinoline alkaloid
78	13.25	691.25579	631.231, 355.129, 335.121, 317.110, 273.122	C_34_H_44_O_15_ [M-H]^−^	n.i.
79	14.381	521.12538	399.089, 152.010, 121.027	C_31_H_22_O_8_ [M-H]^−^	Benzoic acid derivative
80	16.55	493.22444	447.219, 315.179, 161.044	C_21_H_36_O_10_ [M+HCOO]^−^	Geranyl-primeveroside (isomer)
81	17.661	551.33865	505.334, 487.320	C_30_H_48_O_9_ [M-H]^−^	n.i.
82	19.195	675.26108	615.240, 335.121, 317.110, 273.121	C_34_H_44_O_14_ [M-H]^−^	n.i.
83	19.558	711.37422	665.370, 503.317, 179.052	C_36_H_58_O_11_ [M+HCOO]^−^	Modecassic acid hexoside
84	19.886	985.49223	805.430, 643.379	C_49_H_78_O_20_ [M-H]^−^	Methyl-hederagenin (Glc)_2_-glucuronide
85	20.127	1117.53369	955.490, 775.416, 613.367, 179.055	C_54_H_86_O_24_ [M-H]^−^	Ursolic/Oleanolic acid (Glc)_3_-glucuronide
86	20.269	1087.52311	n.d.	C_53_H_84_O_23_ [M-H]^−^	Ursolic/Oleanolic acid Ara-(Glc)_2_-glucuronide
87	20.451	955.48262	775.420, 613.369, 569.380, 179.053	C_48_H_76_O_19_ [M-H]^−^	Ursolic/Oleanolic acid (Glc)_2_-glucuronide
88	20.604	793.43032	631.383	C_42_H_66_O_14_ [M-H]^−^	Ursolic/Oleanolic acid glucosyl-glucuronide
89	20.745	503.31805	n.d.	C_30_H_48_O [M-H]^−^	Madecassic acid (isomer)
90	20.893	793.43112	631.380, 613.368, 569.380	C_42_H_66_O_14_ [M-H]^−^	Ursolic/Oleanolic acid glucosyl-glucuronide
91	20.986	589.31742	545.327, 465.841	C_36_H_46_O_7_ [M-H]^−^	n.i.
92	21.51	809.42556	629.363, 585.384	C_42_H_66_O_15_ [M-H]^−^	Hydroxy-Ursolic/Oleanolic acid glucosyl-glucuronide
93	21.788	955.48179	775.423, 613.368	C_48_H_76_O_19_ [M-H]^−^	Ursolic/Oleanolic acid (Glc)_2_-glucuronide
94	21.949	955.48196	613.368	C_48_H_76_O_19_ [M-H]^−^	Ursolic/Oleanolic acid (Glc)_2_-glucuronide
95	22.002	925.47164	n.d.	C_47_H_74_O_18_ [M-H]^−^	Ursolic/Oleanolic acid Ara-Glc-glucuronide
96	22.132	793.43058	613.367	C_42_H_66_O_14_ [M-H]^−^	Ursolic/Oleanolic acid glucosyl-glucuronide
97	22.176	791.41490	611.353, 523.374	C_42_H_64_O_14_ [M-H]^−^	Moronic acid glucosyl-glucuronide
98	22.337	793.43078	613.369, 569.380, 179.053	C_42_H_66_O_14_ [M-H]^−^	Ursolic/Oleanolic acid glucosyl-glucuronide
99	22.512	793.43059	613.369, 569.378, 179.052	C_42_H_66_O_14_ [M-H]^−^	Ursolic/Oleanolic acid glucosyl-glucuronide
100	22.643	835.44134	655.380, 611.388, 595.359, 483.342, 157.013	C_44_H_68_O_15_ [M-H]^−^	(Acetyl) Ursolic/Oleanolic acid glucosyl-glucuronide
56	22.865	721.35895	675.356, 415.143, 397.132, 277.214	C_33_H_56_O_14_ [M+HCOO]^−^	Digalactosyl-monoacylglycerol-C_18:3_
102	23.033	631.37948	613.374	C_36_H_56_O_9_ [M-H]^−^	Ursolic/Oleanolic acid-glucuronide
103	23.232	631.37960	613.373, 455.350	C_36_H_56_O_9_ [M-H]^−^	Ursolic/Oleanolic acid-glucuronide
61	24.211	571.28370	391.223, 315.044, 255.230, 241.010, 152.994	C_25_H_49_O_12_P [M-H]^−^	lyso-Phosphatidylinositol *C*_16:0_
62	24.921	555.27955	299.041, 255.232, 225.005, 164.984, 152.98337, 125.023, 94.979, 80.963	C_25_H_48_O_11_S [M-H]^−^	Sufoquinovosylmonoacylglycerol-C_16:0_

Peak numbers refer to the number of each chromatographic peak depicted in Figure 1; *t*_R_—relative retention time; n.d.—not detected; n.i.—not identified.

## Data Availability

The original contributions presented in the study are included in the article, further inquiries can be directed to the corresponding author.
